# Photochemical renoxification on commercial indoor photoactive paint

**DOI:** 10.1038/s41598-023-44927-5

**Published:** 2023-10-19

**Authors:** Morgan Vallieres, Stephanie H. Jones, Heather Schwartz-Narbonne, D. James Donaldson

**Affiliations:** 1 Department of Chemistry, University of Toronto, Toronto, Canada; 2 Department of Physical and Environmental Sciences, University of Toronto Scarborough, Toronto, Canada

**Keywords:** Photocatalysis, Atmospheric chemistry, Pollution remediation

## Abstract

Surface chemistry plays an important role in the indoor environment owing to the large indoor surface to volume ratio. This study explores the photoreactivity of surfaces painted with a photoactive paint in the presence of NO_x_. Two types of experiments are performed; illumination of painted surfaces with a nitrate deposit and illumination of painted surfaces in the presence of gaseous NO. For both types of experiments, illumination with a fluorescent bulb causes the greatest change in measured gaseous NO_x_ concentrations. Results show that relative humidity and paint composition play an important role in the photoreactivity of indoor painted surfaces. Painted surfaces could contribute to gas-phase oxidant concentrations indoors.

## Introduction

The high proportion of daily life spent indoors by humans demands a good understanding of indoor chemical processes, which are still poorly characterized^1^, and may differ from those taking place outdoors. One significant difference between indoor and outdoor environments is the very high surface-to-volume ratio of the indoor environment compared to that outside^2^. This higher relative surface area, combined with different indoor vs outdoor chemical sources, may give rise to a different chemical cocktail^1^ of compounds deposited onto indoor surfaces than those outside. Furthermore, the enclosed volume and smaller advection rates indoors can give rise to elevated amounts of directly emitted compounds over levels seen outdoors. One class of compounds highlighted to be of concern in indoor environments is nitrogen oxides (NO_x_)^2–5^. These compounds form an important precursor to ozone and OH concentrations indoors^6^, increasing indoor oxidation capacity^7^.

Evidence suggests that cooking may be at least partially responsible for elevated amounts of NO_x_ measured indoors^8,9^. A study by the World Health Organization^9^ demonstrated elevated production of nitrogen oxide and dioxide from burning events, including use of gas stoves and candles. Once produced, these gases can undergo gas phase and heterogenous chemical reactions^10,11^, some of which may deposit inorganic nitrate onto indoor surfaces. Indeed, a recent study from our group^12^ shows the presence of NO_3_^−^ in “grime films” deposited in the kitchen and living room of an urban apartment. It is possible that gas phase NO_x_ compounds may undergo heterogeneous reactions on indoor surfaces, as is well documented outdoors^13^.

The possibility of photochemistry being important to indoor air chemistry has not received much attention due to the unfavorable illumination wavelengths and low intensities available in most indoor environments. However, it is now well established that chemistry may take place when long wavelength light (λ > 320 nm) interacts with environmental surfaces^10–14^. Heterogeneous photochemical reactions may be initiated by direct absorption of light by a target species, indirectly, via light absorption either by the substrate (i.e., mineral dusts, aromatic films, soot or biologically-derived organic polymers), or by a photosensitizer present with the target species. A recent review^13^ describes the current state of knowledge of heterogeneous photochemistry outdoors. Given the variety of chemical compounds present in the indoor environment and the fact that indoor surfaces are generally illuminated, it seems likely that heterogeneous photochemistry similar to that which occurs outdoors takes place indoors as well.

TiO_2_, a photoactive component of mineral dust^13^, is also an important component of indoor and outdoor paints^15–17^, primarily used to increase the reflection and scattering of light by the paint. Its photocatalytic behaviour has been explored extensively in the context of atmospheric processing of trace gases, such as ozone and nitrogen dioxide^18–22^, but only recently has its reactivity in paint become of interest. Natural mineral dust samples that are known to contain semiconductors, including TiO_2_ and Fe_2_O_3_, have been shown to initiate photo-redox reactions of O_3_ and NO_2_^13,18,23^, as well as several organic compounds^24–26^ following adsorption of the gas phase species to dust surfaces. Light absorption by the semiconductor portions of the dust produces electrons and positively-charged “holes” at the dust surface, which can initiate reduction and oxidation reactions of any species adsorbed there. For example, water adsorbed at the TiO_2_ surface forms OH radicals which may react directly at the surface or may be released into the gas phase when TiO_2_ is photoexcited^27,28^. As well, there is strong evidence that any nitrate anions present at such photoactive surfaces (via deposition of HNO_3_ for example) may be oxidized to NO_3_ radicals or reduced to nitrous acid (HONO) at that surface^16,17^. The possibility that TiO_2_, used as a paint additive, may initiate such redox reactions has inspired its use in some paints as a “green” air cleaner and freshener^29,30^.

In two previous studies^31,32^ we explored whether and how indoor lighting could induce heterogeneous photochemistry of nitrogen oxides on surfaces painted with a commercial indoor white paint; one which is not marketed as being “photoactive”. Although the spectrum of indoor light sources falls at longer wavelengths than those necessary to promote direct photochemistry, the significant presence of TiO_2_ in commercial paints suggests the possibility that heterogeneous photoinduced redox chemistry could occur there, as is known for TiO_2_ in other contexts. In particular, we sought to quantify whether illumination of indoor paint with indoor lighting could give rise to “renoxification”, that is, recycle deposited nitrate anions into the gas phase (as NO + NO_2_ + HONO), and whether such illumination could promote photo-oxidation of gas phase NO to (NO_2_ + HONO). These two chemical processes, if they occur, could alter the local indoor atmospheric oxidation environment, by providing facile sources for photoproduction of ozone (via NO_2_ photodissociation) and OH (via HONO photodissociation), both of which are possible at the wavelengths of indoor lighting. To benchmark our results, we used a 100 W Xe lamp for illumination and TiO_2_ as a substrate. The photochemistry of nitrate anions adsorbed on TiO_2_ and the heterogeneous photoreaction of NO on TiO_2_ have both been reported previously for Xe lamp illumination.

When nitrate anions deposited onto a surface (commercial paint or plain glass slide) were illuminated at 0% relative humidity (RH) using a Xe lamp, we measured significantly more gas phase products (NO + NO_2_ + HONO) when the commercial paint surface was illuminated compared to a plain glass slide surface. The total amount of gas phase species released from the painted surface was about ten times smaller than for a TiO_2_ substrate. The observed product distribution from the painted surface was different from that seen from illuminated TiO_2_ as well: the predominant product from nitrate deposited on the painted surface was NO, whereas from TiO_2_ it was (NO_2_ + HONO). When the painted surface was illuminated with typical indoor fluorescent or incandescent light sources, there was again more gas phase product measured than from nitrate deposited onto a plain glass surface, although the amounts were smaller than those seen using the Xe lamp. Commercial indoor LED or halogen light sources did not induce more gas phase product emission from the painted surface than that obtained from the nitrate on plain glass. We rationalized these findings as being reflective of the overlap between the various lamp emission spectra and the absorption spectrum of TiO_2_, although the reason for the different product distribution obtained from the painted surface vs. the TiO_2_ remains unknown.

When we exposed the painted substrate (with no nitrate present) to gas phase NO, a small loss of NO to the substrate was measured in the dark, at both 0% RH and 50% RH. No photoenhancement of the uptake was measured at either humidity, and no production of (NO_2_ + HONO) was seen in the dark, or under illumination from Xe, fluorescent or incandescent sources. This finding is in contrast to what is seen when a TiO_2_ substrate is illuminated with the Xe lamp: a significant loss of gas phase NO and release of (NO_2_ + HONO) into the gas phase are recorded, in excellent agreement with previous reports.

Taken together these results are a bit perplexing. On the one hand, there is evidence for the photocatalysed reduction of nitrate anion (presumably by TiO_2_) on the painted substrate illuminated by Xe, fluorescent of incandescent lights, albeit with a somewhat different distribution of gas phase products than obtained from pure TiO_2_ under Xe lamp illumination. On the other hand, there is no heterogeneous photochemistry of NO observed using the same substrate, with any illumination source. These findings and this mystery motivated the present study, where we consider a different commercial paint: one that is specifically marketed as being “photoactive” (PA).

PA paints are designed to optimize the “air-cleaning” properties (i.e., removal of VOCs) under indoor illumination. This enhanced heterogeneous photochemical ability suggests that there may be significant differences between the renoxification arising from substrates painted with PA paint vs. those painted with non-photoactive (NPA) paints. A small amount of work has been done exploring this point, mostly looking at NO_2_ removal by such paints^33,34^. Indeed, as reported in these studies, PA paints are very effective at reducing indoor NO_2_. Although there was some discussion of the production of VOCs from the binder used in PA paint, reaction products arising from the removal of NO_2_ were not discussed. Here we investigate the role of PA paint in renoxification indoors by conducting the same experiments as previously performed with NPA paint^31,32^ i.e. illumination of PA paint with nitrate ions deposited on the surface and illumination of PA paint in the presence of NO to allow a direct comparison of the renoxification potential between different paints. As our point is to compare properties of representative commercial indoor paints, no analysis was done of the chemical components in either paint. As well, we report here the effect of increasing the RH on the photochemistry of nitrate deposited on the NPA paint.

## Experimental details

The experimental methods have been described fully in our earlier publications^31,32^. Briefly, painted sample substrates (glass slides) were placed into in a ~ 250 mL stainless steel chamber and illuminated from above through a quartz window by a light source of interest. Gases flowing through the chamber were directed into a commercial chemiluminescent NO_x_ analyser (Teledyne T200U) that recorded changes in the concentrations of gas phase NO_x_ when the sample was illuminated vs. when it was in the dark. Our previous work identified that three illumination sources were most effective at promoting chemistry on NPA paint. These were used here as well: a 150 W Xe arc lamp, which simulates solar illumination, and commercially-obtained indoor fluorescent and incandescent bulbs. Their emission spectra are shown in both earlier works^31,32^.

As the substrate, we used a commercially available paint, *Fresh Air* from Climasan StoColor (Produktblatt StoColor Climasan Master (sto-sea.com)/last accessed Sept 27, 2023), as the photoactive agent. This paint is marketed as being designed to eliminate room odors and break down airborne harmful substances in the presence of indoor lighting. As reported in our previous works^31,32^, the NPA paint we used was also commercially available, BEHR MARQUEE interior semi-gloss ultra-pure white enamel paint. Both paints used here are proprietary formulations, so the composition of the binding agents is unknown to us.

The NO_x_ detector utilized in this work operates by quantifying the NO amount in an air sample via the chemiluminescence emitted from the excited NO_2_ product of the NO + O_3_ chemical reaction. The chemiluminescence intensity is measured first by adding ozone to the air sample of interest, then a second reading is performed with the air sample diverted over a heated Mo surface, which catalytically converts NO_2_ (and some other species, such as HONO) to NO prior to adding ozone. The difference between the two readings is taken as indicating the NO_2_ amount in the air sample and their sum is the measured NO_x_. Of particular importance to the present work, HONO will be reported with NO_2_ as the NO_2_ amount. In Schwartz-Narbonne et al.^32^ we used a denuder to remove HONO prior to entering the NO_x_ detector, in order to establish a true “NO_2_” reading, but this was not used here. Therefore, in the following we report NO and (NO_2_ + HONO), and sum the separate NO_2_ and HONO amounts reported in reference 32 for comparison between the PA and NPA results.

We used window glass cut into 4 × 2 cm segments as substrate. These were cleaned using a commercial cleaning agent, rinsed with ultrapure water, then wiped with Kimwipes and MeOH at least 3 times. Each substrate plate was then painted with an even, thin coat of the photoactive paint and left to activate in a window sill for 4–6 weeks before use.

Two types of experiments were conducted. The first focused on heterogeneous nitrate (NO_3_^−^) photochemistry on painted substrates and the second examined the interaction of gaseous NO with painted substrates. Experiments of the first type were performed on painted substrates with a deposit of inorganic nitrate evaporated onto the surface. A 0.25 M solution of NaNO_3_ was prepared and 0.3 g of this was weighted onto each painted sample and evenly coated, ensuring no runoff. The sample was then placed on a hot plate and covered with aluminum foil in a dome shape, in order to protect it from laboratory lights. The sample was left to dry for approximately 10 minutes at the lowest possible hot plate setting. Once the sample was dried, it was removed from the hot plate, covered and left to cool to room temperature. A flow of zero air (0% RH or 50% RH) was passed over the sample and directed into the NO_x_ analyser as samples were successively illuminated and darkened.

For the NO experiments, the painted substrate was exposed to a flow of zero air mixed with NO in N_2_ which was directed into the chamber as samples were successively illuminated and darkened. For all experiments at 0% RH, zero air (or zero air with NO) was directed to the reaction chamber directly, whereas for experiments at 50% RH, the zero air was humidified to 50% RH by directing some of the flow through a water bubbler. Each experiment was conducted twice for each light source studied, with a total of 3 illumination on/off cycles. The total release of NO_x_ shown in the figures was determined by the difference of observed NO_x_ when the light was on versus off.

## Results

### NO_x_ emission from illuminated nitrate-doped paint

**Figure 1:**
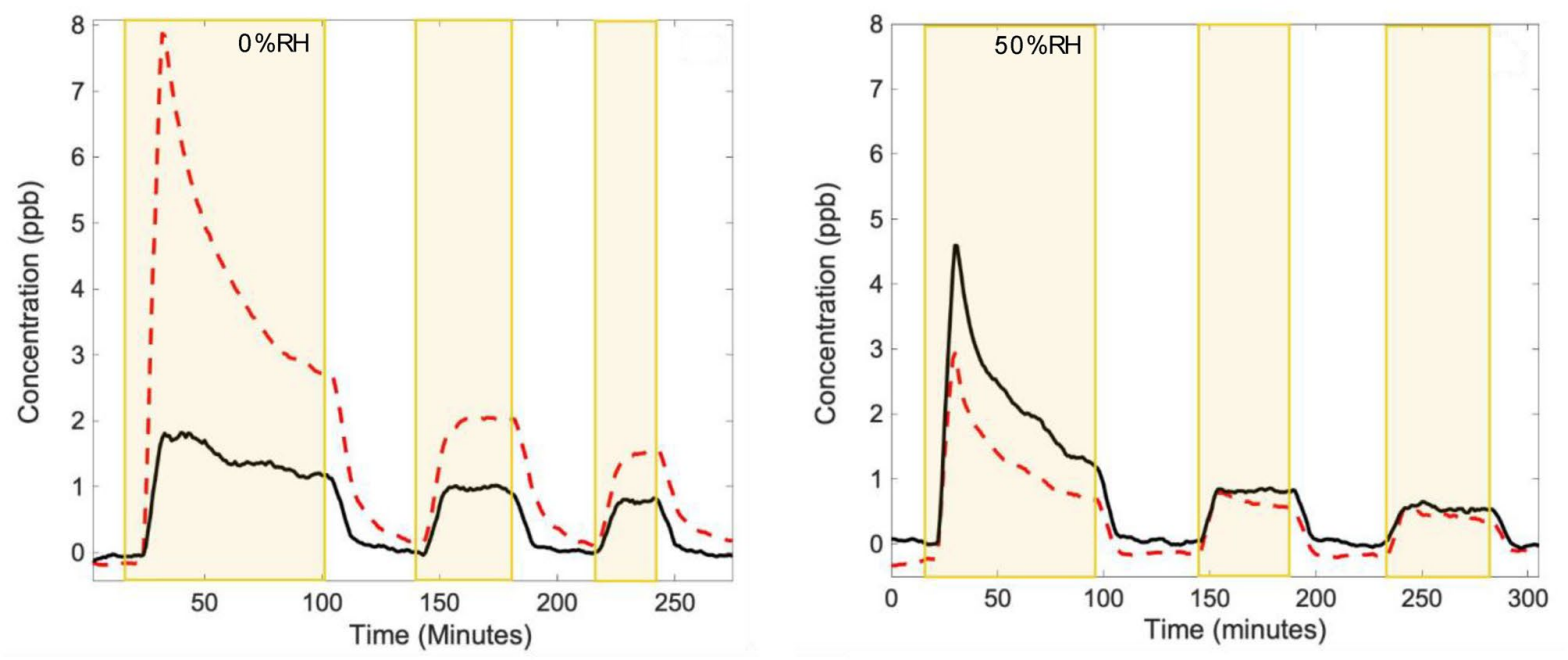
Emission of NO (red dashed line) and (NO_2_ + HONO) (black solid line) when nitrate-doped PA paint is illuminated with fluorescent light (yellow shading) and in the dark (no shading) for 3 cycles at 0% (left frame) and 50% (right frame) relative humidity.

Figure 1 shows typical product emission as a function of time for nitrate-doped PA painted substrates at 0 and 50% RH under illumination by a fluorescent bulb. Illumination by the two other sources gives similar results. At both 0 and 50% RH, the initial exposure of the sample to the light, shown by the yellow shading, gives an intense burst of NO_x_. Following this initial burst, the amount released diminishes and become stable with repeated illumination cycles, as shown in the later time white and yellow shaded zones. Increasing the RH to 50% from 0% results in a decrease in the released NO and an increase in (NO_2_ + HONO) compared to that observed at 0% RH.

**Figure 2:**
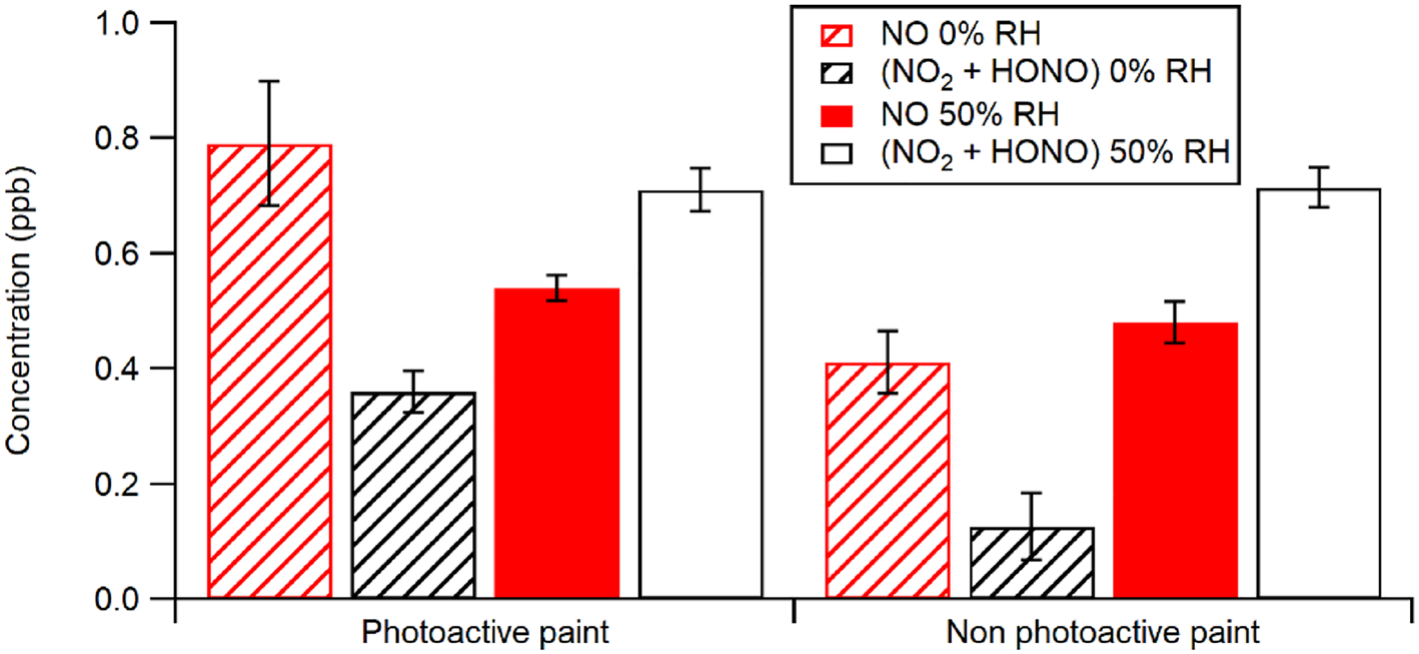
Comparison of gas phase products seen from illumination of PA and NPA nitrate doped indoor white paint illuminated with a Xe lamp at 0% RH and 50% RH. The error bars on these figures were determined by the standard deviation of the observed NO_x_ from each illumination on/off cycle once the emissions were stable.

Figure 2 summarizes and contrasts the differences at 0% and 50% RH for NO_x_ emissions observed from the illumination of nitrate doped PA and NPA paints with a Xe source. The concentrations are taken at steady state after the concentrations had stabilized (that is, excluding the initial burst observed during the first illumination cycle). For both paints, the product distribution at 0% RH favours NO over (NO_2_ + HONO). At 50% RH there is a marked increase in (NO_2_ + HONO) compared to what is observed at 0% RH for both paints, with only small changes seen in the NO. This shifts the product distributions observed with both paint types at the higher RH, suggesting that co-adsorbed water may be playing a role in the surface reaction.

**Figure 3:**
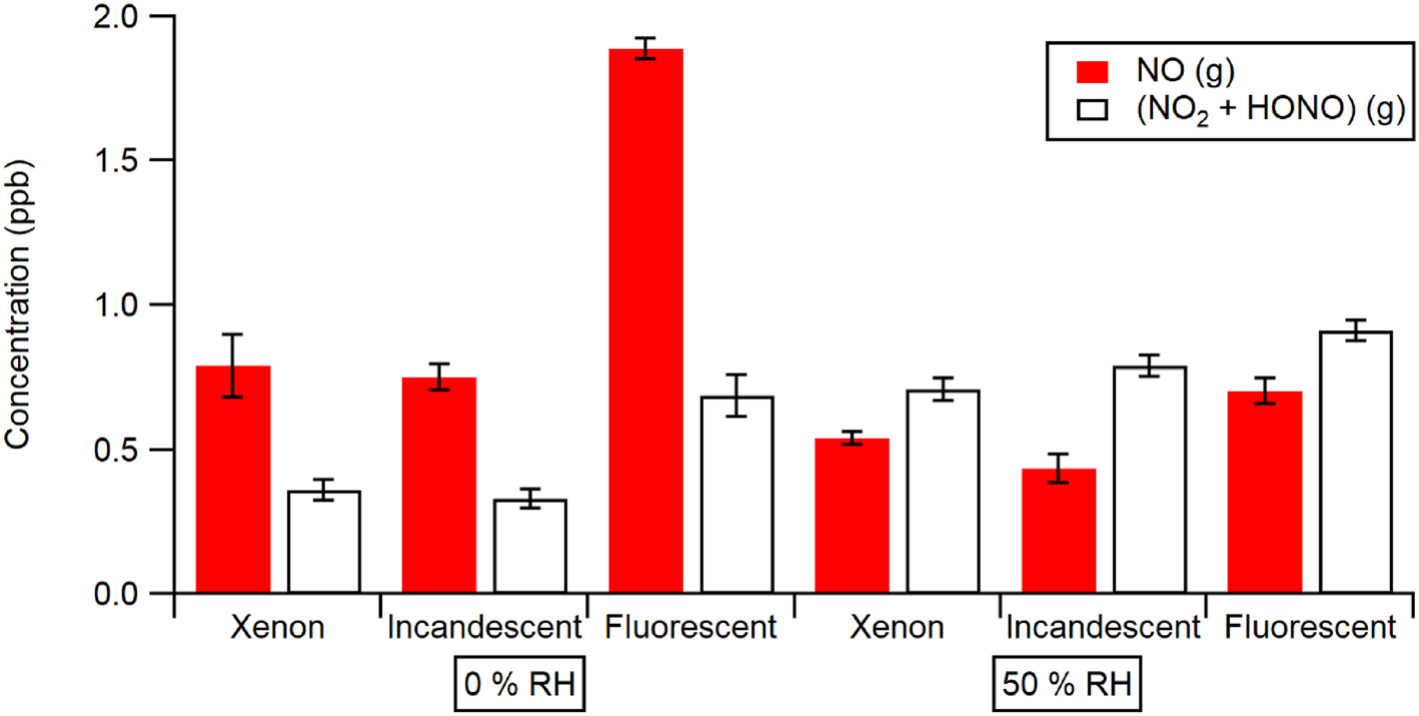
Observed NO_x_ emissions from illumination of nitrate doped PA paint at 0 and 50% RH by three different light sources (Xenon, incandescent and fluorescent). The error bars on these figures were determined by the standard deviation of the observed NO_x_ from each illumination on/off cycle once the emissions were stable.

Figure 3 contrasts the observed NO_x_ emission from nitrate doped PA paint illuminated with different light sources at 0% and 50% RH. In this figure as well, the results are quantified after the initial burst of gases, when repeated illumination gives steady concentrations. In all cases the main product is NO at 0% RH; this changes to (NO_2_ + HONO) at 50% RH. The largest emissions of NO_x_ at both humidities are observed under illumination from a fluorescent lamp, with xenon and incandescent sources yielding less gas phase product. This observation contrasts with that made during illumination of nitrate doped NPA painted surfaces, where the Xe lamp gives the greater product yield^32^ by a factor of 2–3 compared to the other light sources. The total NO_x_ emissions (NO + (NO_2_ + HONO)) registered from both paint types were similar using the Xe lamp at both 0% and 50% RH. For the PA paint, the incandescent lamp gave a similar total emission to that seen with the Xe lamp at both RH values. Under fluorescent lamp illumination, however, increasing the RH to 50% caused an overall decrease in the total product emission, mainly due to the drop in the NO amount.

To our knowledge, there have been no studies or reports of NO_x_ emission from painted surfaces that do not have any nitrogen oxide deposits. (Note that indoor surfaces will contain a deposit of nitrate from both advection from outdoors and from indoor activities, such as cooking^12^). For this reason, we did not previously^32^ illuminate the NPA painted substrates in the absence of a nitrate deposit. As the point of the present work is to compare the PA and NPA results under the same conditions, we did not measure NO_x_ emissions from PA paint with no nitrate deposit either.

### Interaction of NO_(g)_ with illuminated indoor paint

**Figure 4:**
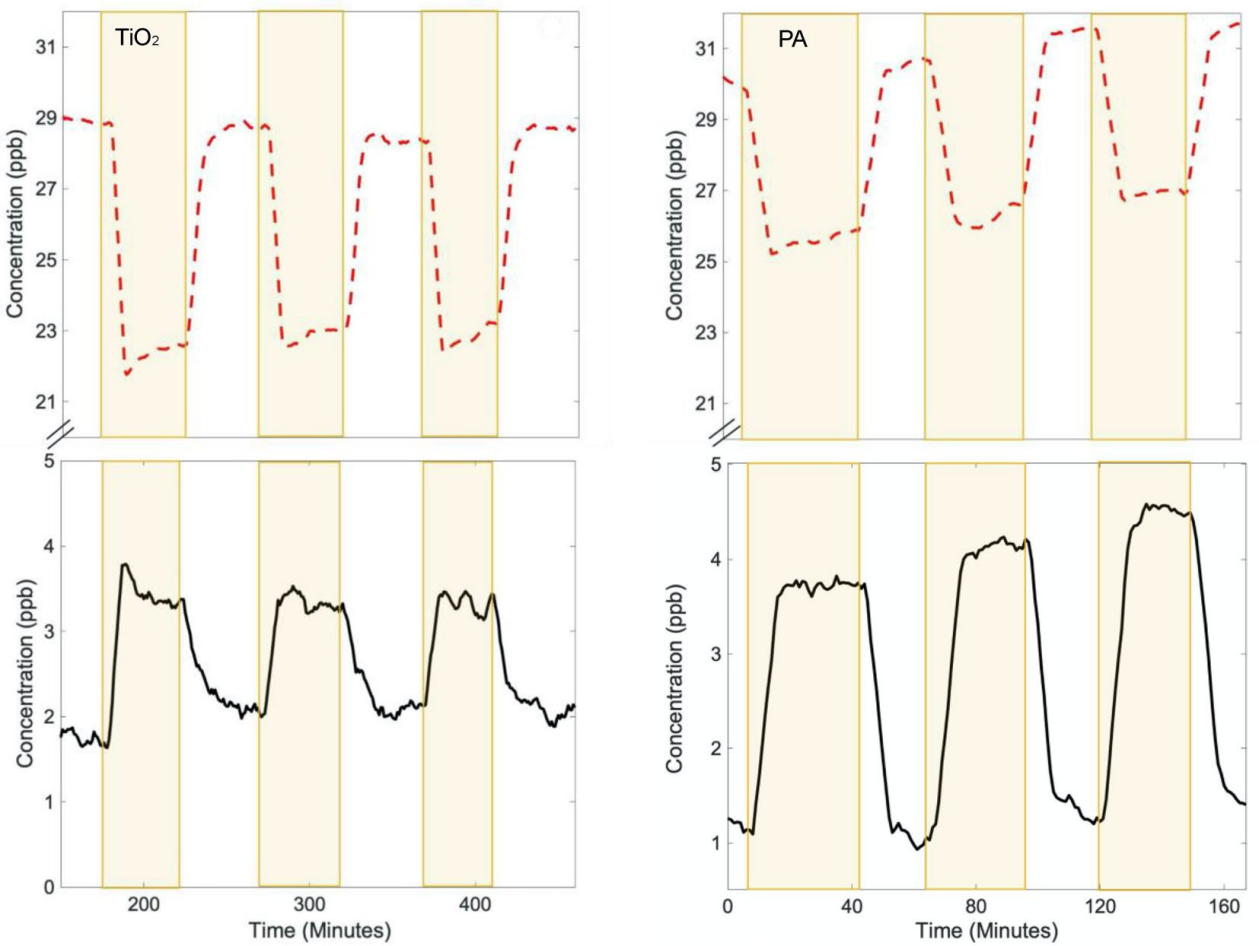
Uptake of NO (upper red dashed line) and prompt release of (NO_2_ + HONO) (lower black trace) when 1.0 g of TiO_2_ (left panels) and a PA painted substrate (right panels) are illuminated with a Xe lamp at 50% RH. The TiO_2_ results are those from reference 31. Yellow shading indicates illumination periods. The NO_x_ amounts are given by the sum of the NO and (NO_2_ + HONO) amounts shown.

The interaction of NO_(g)_ with illuminated TiO_2_ has been well studied and gives rise to loss of NO from the gas phase, with a commensurate formation of NO_2_ as well as some surface nitrate^35^. This result is illustrated in the left panel of Fig. 4 for NO interacting with TiO_2_ under illumination by a Xe lamp. A loss of about 20% of the gas phase NO, with an approximate 25% conversion to (NO_2_ + HONO) is seen at 50% RH, similar to earlier reports^19^. The observations are similar when PA paint is illuminated in the presence of approximately 30 ppb NO: upon illumination, there is an immediate loss of NO and a corresponding gain in (NO_2_ + HONO). The illuminated paint gives rise to a somewhat smaller loss of NO (~ 15%), but a much larger conversion to (NO_2_ + HONO): about 50–60% of the NO is emitted as gas phase product.

**Figure 5:**
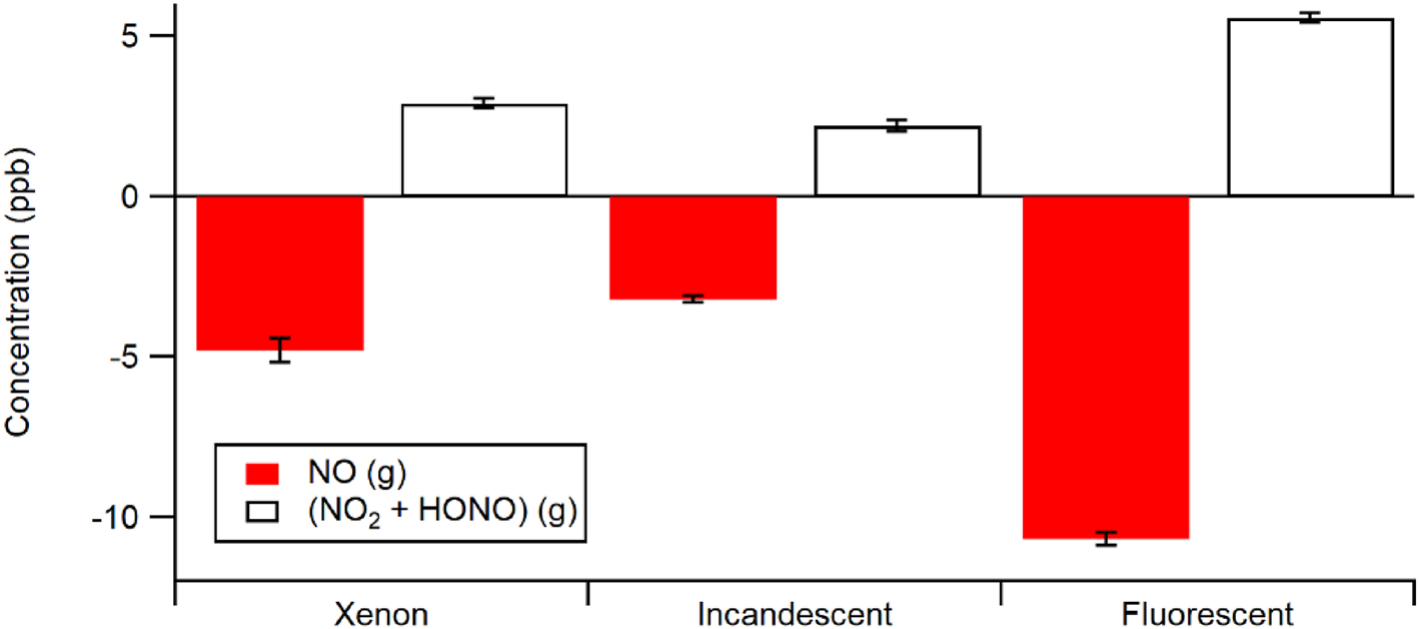
Summary of results at 50% RH for loss of NO and production of (NO_2_ + HONO) when gas phase NO is exposed to PA painted substrates and illuminated by three different light sources. The error bars on these figures were determined by the standard deviation of the observed NO_x_ from each illumination on/off cycle once the emissions were stable. Note that the total change in NO_x_ is given by the sum of the ∆NO and ∆(NO_2_ + HONO) amounts.

The same overall result is obtained independent of the illumination source used (Xe, fluorescent or incandescent lamp) for PA painted substrates as shown in Fig. 5 at 50% RH. The largest effect on NO_x_ emission is observed for illumination from the fluorescent lamp: ~ 33% loss of NO and ~ 50% conversion to NO_2_ was obtained. The incandescent lamp gave rise to a ~ 10% loss of NO from the gas phase, with a ~ 65% conversion to gas phase products. Although the fraction of gas phase NO lost is similar for the PA paint under any of the lamps as that seen on TiO_2_ under Xe illumination, the conversion ratios on the PA paints are considerably greater than the approximately 25% seen with the TiO_2_ substrate under Xe illumination. These observations are all vastly different to what is observed with NPA paint, which showed significant loss in the dark but very little loss or conversion of NO on its surface upon illumination.

## Discussion

As mentioned in the Introduction, we have previously reported that indoor surfaces (especially kitchen surfaces) have significant amounts of inorganic nitrate as a deposit^12^. This deposit may arise from heterogeneous reaction of ambient NO or via advection and deposition of particulate nitrate, HNO_3_ or N_2_O_5_ from outdoor air. In Schwartz-Narbonne et al.^32^ we demonstrated that NPA paints can act as substrates for photoactivation of this nitrate under indoor illumination. Although this process is not highly efficient on NPA paints under indoor lighting at 0% RH, it becomes more so at 50% RH (as illustrated in Fig. 2). Using PA paint, the release of gas phase products from deposited nitrate is similar to or somewhat greater than that from NPA painted surfaces under any of the illumination sources explored here. Notably, with both PA and NPA paints, increasing the RH from 0 to 50% changes the product distribution significantly, away from NO as the primary product to (NO_2_ + HONO) and therefore enhances the overall emission of photochemically active species, thus potentially affecting the indoor atmospheric oxidative environment. Although this RH dependence in product distribution differs from that reported by Ndour et al.^23^ for nitrate-doped TiO_2_ illuminated by 330–420 nm UV lamps, it is consistent with a recent report by Ma et al.^36^, who report an increase in both NO_2_ and HONO production rates from nitrate-doped TiO_2_ illuminated using similar UV lamps. An enhancement in these more oxidized products is likely due to the influence of adsorbed water on the photochemistry, perhaps driven by the known production of OH from illuminated water adsorbed on TiO_2_^13^.

To our knowledge, there are no previous reports of the heterogeneous photochemistry of NO on a PA painted substrate. There have been a handful of studies that have looked at the interaction of NO with a TiO_2_ surface under illumination^35,37–39^. Work by Angelo et al.^39^, demonstrates that the ambient RH does not play a strong role in the heterogeneous photooxidation of NO on TiO_2_. The results of Devahasdin et al.^38^ show a ~ 30% conversion efficiency of NO to NO_2_, similar to what we observed with TiO_2_. Interestingly, Monge et al.^38^ report a significant production of ozone from the photoreaction of NO on TiO_2_ and propose that it arises from photochemistry of nitrate anions formed on the surface as an initial product of the NO surface chemistry. Topalov et al.^37^ investigated the relative efficiencies of some indoor light sources on NO abatement using a TiO_2_-coated wallboard substrate. They also report a modest (~ 15%) conversion of NO to NO_2_, in keeping with our measurements. We note that in those experiments, similar to those we report here, the measured “NO_2_” is also likely to be (NO_2_ + HONO).

The present results illustrate that a commercially available PA paint, illuminated by indoor lighting sources, shows a similar loss of gas phase NO as observed with TiO_2_ substrates, but a much higher conversion to (NO_2_ + HONO)—well over 50% under the experimental conditions studied here, compared to ~ 25% for TiO_2_ substrates. The most efficient photochemical loss of NO on the PA paint was obtained using fluorescent lighting at 50% RH. Illumination with incandescent or xenon lamp sources gave smaller photochemical uptake of NO than seen using the fluorescent source, but similar high conversion to (NO_2_ + HONO). All of these results are markedly different from what is obtained using NPA paints, where there is no photochemistry of NO observed^31^. The very high NO to NO_2_ conversion efficiency exhibited by the PA paint may have real implications for the indoor oxidizing environment, as the photolysis of both NO_2_ and HONO (to form ozone and OH, respectively) is facile under indoor lighting conditions. It is particularly worth noting that the total flux of NO_x_, as given by the sum of the positive flux of (NO_2_ + HONO) and the negative flux of NO shown in Fig. 5, is zero or quite small for simulated sunlight (i.e., Xe lamp) or incandescent illumination, but shows a clear net loss to the painted surface under fluorescent lighting. If generally true for other commercial PA paints, this suggests that the air-cleaning ability is quite dependent on the type of indoor illumination. This should be factored into a choice of paint for indoor air purification purposes.

## Conclusions

The results outlined above indicate that predicting the photo-reactivity of paints towards certain molecules—in this case NO_x_—using TiO_2_ as a proxy for the photoactive agent in paint is not always justified. Even though TiO_2_ is a significant component of both white NPA and PA paints, significant differences are observed in the heterogeneous photochemistry of both adsorbed nitrate and gas phase NO between painted surfaces and TiO_2_ alone. Furthermore, differences are also seen between the paints investigated here, taken to be representative of commercial indoor photoactive and non-photoactive paints. For both types of experiments conducted in this study, illumination of PA paint by a fluorescent bulb was the most effective at initiating heterogeneous photochemistry; notably more effective than illumination by a Xe lamp that has much better overlap with the TiO_2_ absorption spectrum. This is in keeping perhaps with the design of the photoactive paint for indoor air cleaning use. The present work has demonstrated that painted indoor surfaces, especially surfaces painted with a PA paint, have the potential to contribute to indoor gas-phase oxidant levels under conditions of illumination and humidity expected indoors.

## Data Availability

All data generated or analysed during this study are included in this published article.
